# Modulation of Indoleamine 2,3-Dioxygenase 1 During Inflammatory Bowel Disease Activity in Humans and Mice

**DOI:** 10.1177/11786469231153109

**Published:** 2023-02-09

**Authors:** Elisa Proietti, Renske W.M. Pauwels, Annemarie C. de Vries, Elena Orecchini, Claudia Volpi, Ciriana Orabona, Maikel P. Peppelenbosch, Gwenny M. Fuhler, Giada Mondanelli

**Affiliations:** 1 Department of Experimental Medicine, University of Perugia, Italy; 2 Department of Gastroenterology and Hepatology, Erasmus MC, Rotterdam, The Netherlands

**Keywords:** Indoleamine 2,3 dioxygenase-1, IBD, colitis, Ustekinumab, Vedolizumab, N-acetyl serotonin

## Abstract

**Background and Aims:**

Indoleamine 2,3 dioxygenase-1 (IDO1), a key enzyme in tryptophan metabolism, is strongly up-regulated both in human inflammatory bowel disease (IBD) and animal models of colitis, however its role in the pathogenesis is still controversial. In this study, we investigated IDO1 expression and activity in a mouse model of DSS-induced chronic colitis as well as in colon biopsies and sera from IBD patients.

**Methods:**

Chronic colitis was induced in mice through the oral administration of dextran sodium sulfate (DSS), and IDO1 activity was induced by i.p. treatment with N-acetyl serotonin (NAS). IDO1 expression and catalytic activity (measured as Kyn/Trp ratio) was evaluated in sera and tissue samples collected from mice and 93 IBD patients under immunotherapy with Vedolizumab (VDZ) or Ustekinumab (UST).

**Results:**

Strong up-regulation of IDO1 was found in colons of mice with acute colitis, which follows disease activity. Enhanced IDO1 activity by NAS treatment protects the intestinal mucosa during the recovery phase of chronic colitis. In IBD patients, IDO1 expression and activity correlate with the severity of mucosal inflammation with inflamed regions showing higher IDO1 expression compared to non-inflamed regions within the same patient. Endoscopic response to VDZ/UST treatment is associated with decreased expression of IDO1.

**Conclusions:**

This is the first study demonstrating immunomodulatory activity of IDO1 in a chronic mouse model of DSS-induced colitis. As its expression and catalytic activity correlate with the grade of mucosal inflammation and treatment response, IDO1 could represent a promising biomarker for disease severity and treatment monitoring in IBD.

## Introduction

Inflammatory Bowel Disease (IBD) is the term used to define chronic inflammatory disorders affecting the intestine, which include Crohn’s disease (CD) and ulcerative colitis (UC). These pathologies are generally characterized by the alternation of periods of remission with acute inflammatory phases, which manifest with symptoms such as severe diarrhea, abdominal pain, fatigue, flatulence, blood in stool, and weight loss.^1^ Substantial progress in our understanding of IBD pathogenesis has been achieved during the past years, which has resulted in the development of several targeted therapeutic agents, including biologicals directed at TNFα, IL12/IL23, and the α4β7 integrin, as well as small molecules targeting the JAK kinase pathway. However, as a large proportion of patients does not respond or loses response to currently available treatment options, novel avenues of intelligent drug design are still under investigation.

Several lines of evidence suggest a role for l-Tryptophan (Trp) metabolism in IBD pathogenesis.^2-5^ Trp is an essential aromatic amino acid and its metabolism follows 3 major pathways in the gastrointestinal tract: the kynurenine (Kyn) pathway in both immune and epithelial cells, the serotonin pathway in enterochromaffin cells and the remaining amount of Trp is metabolized by the gut microbiota into indole and its derivatives.^6^ Trp metabolism generates several bioactive compounds that regulate a variety of physiological and pathological processes, including cell growth, metabolism, emotions, and immunologic responses.^7^

The Kyn pathway is the most prominent route of Trp metabolism. Indoleamine 2,3-dioxygenase 1 (IDO1) is the metabolic enzyme that catalyzes the first and rate-limiting step of this pathway, resulting in a series of immunoactive metabolites collectively known as kynurenines.^8-10^ IDO1 is induced by pro-inflammatory cytokines (including interleukin (IL)-1β, IL-6, IL-18, tumor necrosis factor α (TNF-α), and interferon-γ)^11^ and through its catalytic activity plays a critical role in regulating immune responses. It was demonstrated that Kyn acts as an agonist of the transcription factor Aryl-hydrocarbon Receptor (AhR)^12^ which mediates immune-regulatory responses driving naive CD4+ T cells toward the conversion into immunosuppressive Treg cells rather than pro-inflammatory T-helper 17 (Th17) cells, selectively inducing apoptosis of thymocytes as well as T-helper 1 (Th1) cells, and favoring the production of anti-inflammatory cytokines.^13,14^ IDO1 is also endowed with signal-transducing functions, which are independent of its enzymatic activity and rely on 2 immunoreceptor tyrosine-based inhibitory motifs (ITIMs) present in the non-catalytic domain of the protein.^15,16^ As a result, IDO1 controls and fine-tunes both innate and adaptive immunity under a variety of conditions, ranging from pregnancy and transplantation to infection, chronic inflammation, autoimmunity, and neoplasia.^5,17-19^ Over the past years, given the great importance of IDO1 in the regulation of immunity, several researchers have worked to find molecules capable of modulating the expression and activity of IDO1.^20^ Recently, Mondanelli et al^21^ suggested that N-acetylserotonin (NAS), a Trp metabolite generated from serotonin, can directly bind IDO1 on a newly identified allosteric pocket and thus acts as a positive allosteric modulator (PAM) of IDO1 both in vitro and in vivo resulting in an increased production of Kyn.

Despite in-depth knowledge of the immune-modulatory activity of IDO1, the role of this enzyme in IBD remains controversial. In the homeostatic state, gut expression of IDO1 is low and mostly occurs in cells of the lamina propria, but in the inflamed mucosa observed during disease activity, its expression increases.^22^ Several studies in patients affected by CD or UC showed increased expression of IDO1 mRNA and protein in both the lamina propria and epithelium and an increased serum Kyn/Trp ratio in patients with active disease compared to inactive disease or healthy controls,^3,23,24^ raising questions regarding the function of this enzyme in the gastro-intestinal tract.

To elucidate the role of IDO1 in IBD, several studies were performed using different mouse models of colitis, leading to contradictory results.^25-31^ In accordance with its generally assumed immunomodulatory activity, several reports have highlighted the protective role of IDO1 in animal models of colitis induced by 2,4,6-trinitrobenzene sulfate (TNBS) or dextran sodium sulfate (DSS), concluding that IDO1 upregulation represents a counter-regulatory mechanism to combat the inflammatory process.^25-27,29,32,33^ However, other studies have shown that IDO1-deficient mice are protected from colitis and that inhibition of IDO1 enzymatic activity results in an improvement of disease symptoms,^28,30,31^ thus questioning the role of IDO1 in colitis. Besides the different mouse models used, most of these studies were conducted in acute colitis models, and thus mechanistically far removed from the chronic phenotype of CD and UC with their intermittent disease flares. Here, we investigated IDO1 expression and activity in a mouse model of DSS-induced chronic colitis as well as in colon biopsies and sera from IBD patients. Our data indicated that (*i*) after an initial increment of IDO1 expression in the acute inflammatory phase, the protein levels and the enzyme activity drop during the milder chronic phase of the disease; (*ii*) mice treated during the chronic phase with NAS show improved mucosal healing; (*iii*) IDO1 expression and activity correlate with the severity of mucosal inflammation in patients with IBD; (*iv*) the response to VDZ and UST treatment is associated with a reduction of intestinal level and systemic activity of IDO1 in IBD patients. Together, our data suggest that IDO1 activity has a positive modulatory effect on disease activity and follows disease activity state.

## Methods

### Murine model of DSS-induced chronic colitis and treatment

**Figure 1.**
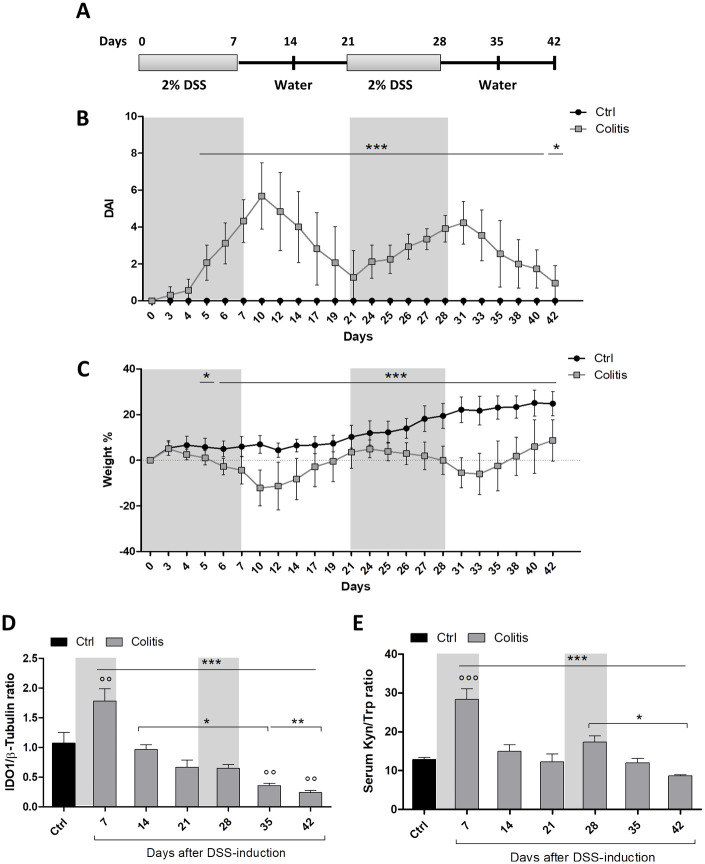
IDO1 expression and catalytic activity follow disease activity during acute and chronic phases of disease: (A) scheme of chronic DSS colitis induction in mice: mice in the colitis group were subjected to alternating cycles of 2% DSS solution and normal drinking water, while control mice received normal drinking water only, (B) disease activity index (DAI) of DSS-treated mice and controls was daily measured by combining scores of weight loss, stool consistency, and presence of fecal blood, (C) weight change of DSS-treated mice and controls over time was calculated as a percentage of initial weight using the formula: [(Weight at day X/Weight at day 0) × 100]. Data in B and C are presented as the mean ± SD from 15 mice/group of 3 independent experiments. (D) Lysates from colons of DSS-treated and control mice were analyzed by Western blot analysis using an antibody against IDO1. Anti-β-tubulin antibodies were used to correct for total protein content. Densitometric analysis of IDO1 and β-tubulin ratios is reported as mean ± SD from 9 mice/group (3 for each time point of 3 independent experiments), (E) serum samples of DSS-treated mice and controls were analyzed for kynurenine (Kyn) and Tryptophan (Trp) levels by HPLC. Results are reported as the Kyn/Trp ratio ± SD from 9 mice/group (3 for each time point of 3 independent experiments). Two-way ANOVA, followed by post hoc Bonferroni test was used for the analysis of B-E. Ctrl, control mice receiving normal drinking water. Colitis, mice subjected to alternating cycles DSS solution. °°*P* < .01 and °°°*P* < .001 versus control group. *P < .05, **P < .01, and ***P < .001 versus selected groups.

To reproduce the chronic nature of IBD, we employed a chronic DSS-colitis model, in which female C57BL/6 mice were provided with a solution of filtered water containing 2% DSS w/v (*Sigma-Aldrich*) ad libitum over a 7-day period (cycle 1). After the first induction cycle, DSS-solution was replaced with normal drinking water for 14 days (recovery phase), followed by a second DSS challenge (cycle 2). At the end of cycle 2, mice were given normal drinking water for a further 14 days (Figure 1A). Over the course of the experiments, mice receiving normal drinking water were used as control. The in vivo experiments performed in this work were approved by the Italian Ministry of Health.

Colitis severity was evaluated by daily monitoring of mice, as previously described.^30,34^ Specifically, changes in body weight, stool consistency, and presence of fecal blood were scored as follows:

- weight loss (0 points = no weight loss or weight gain; 1 points = 5%-10% weight loss; 2 points = 11%-15% weight loss; 3 points = 16%-20% weight loss; 4 points >21% weight loss); body weight change was calculated as a percentage of initial weight using the formula:

$$\%\;weight\;change\;=\;\left[\left(\begin{array}{l} Weight\;at\;day\;X\;/\\ \;Weight\;at\;day\;0 \end{array}\right)*100\right]$$

- stool consistency (0 points = normal and well formed; 1 point = soft and sticky; 2 points = diarrhea);
- bleeding stool score (0 points = no blood; 1 point = blood in stool or around the anus; 2 points = bleeding anus).

The sum of mentioned parameters constitutes the disease activity index (DAI) and was measured from day 0 to day 42.

Mice were treated i.p. with NAS (10 mg/kg), 3 times per week, starting from the first recovery period at day +12.

### Mouse sample collection

Mice were sacrificed at day 7, 14, 21, 28, 35, or 42 after DSS-colitis induction. Serum was collected and stored at −80°C until analysis. The entire colon was quickly removed and colon length was determined as a marker of inflammation. After removing the stools using tweezers, pieces of colon were cut, fixed in 10% phosphate-buffered formalin, embedded in paraffin and sectioned at 5 μm for immunohistochemistry (IHC). Serial sections were prepared and stained with hematoxylin and eosin for histologic grading. The remaining part of the colons were sliced longitudinally and washed several times with a phosphate-buffered saline (PBS) solution. A fragment of each colon was homogenized in 500 µl of PBS containing protease inhibitors using a grinder. The homogenized colon was vortexed for 30 seconds and then centrifuged for 20 minutes at 13 000 rpm. Supernatant was harvested and protein quantification was performed using the Bradford assay (Biorad^®^).

### Patient cohort and outcome definitions

Adult patients with UC, CD, and IBD-unclassified (IBD-U) who started VDZ or UST between December 2016 and November 2018 at the Erasmus MC University Medical Center Rotterdam were prospectively included after obtaining written informed consent. The study protocol was approved by the Medical Ethics Committee (METC) Rotterdam (MEC 2004-168 2012), the Netherlands. Inclusion criteria were active endoscopic disease at baseline, defined as a Mayo endoscopic score of ⩾1, simple endoscopic score for (SES) CD ⩾3 or Rutgeerts’ score ⩾2. The concomitant use of corticosteroids and immunomodulators (IM) was allowed. VDZ induction therapy consisted of four 300 mg infusions for UC patients and was scheduled at baseline (week 0), week 2, week 6, and week 14. All CD patients received an additional VDZ infusion at week 10. As maintenance therapy, an intravenous infusion of 300 mg VDZ every 8 weeks was scheduled. UST induction therapy (UC patients only) consisted of 6 mg/kg intravenously and maintenance therapy consisted of subcutaneous injections of 90 mg UST every 8 weeks.

Follow-up endoscopies for the assessment of therapy response and collection of sera and biopsies were performed after 16 weeks. Clinical and demographic characteristics (age, gender, smoking status, disease characteristics, and treatment history) were collected at baseline. Fecal calprotectin (FC) was determined using a quantitative enzyme linked immunosorbent assay (ELISA) (Bühlmann Laboratories AG, Schönenbuch, Switzerland) or QuantOn cal (QoC) (Preventis, Germany) FC home test. Severity of disease was classified as depicted in Table 1. Simple clinical colitis activity index (SCCAI, for UC patients) and Harvey-Bradshaw index (HBI, for CD patients) were used to define the clinical disease activity. Endoscopic inflammation was determined using the Mayo endoscopic score for patients with UC, the simple endoscopic score (SES) in patients affected by CD, and the Rutgeerts’ score for postoperative CD patients. In patients with an ileostomy, ileoanal pouch anastomosis or ileorectal anastomosis, endoscopic disease activity was classified as no, mild, moderate, or severe as judged by the endoscopist. Grade of inflammation at histological examination was classified by a blinded pathologist on a four-point scale: 0, no active histologic disease activity; 1, mild active inflammation (cryptitis, but no crypt abscesses); 2, moderate active inflammation (few crypt abscesses); 3, severe active inflammation (numerous crypt abscesses).

**Table 1.** Schematic overview of categorization of disease activity.

Grade of inflammation	SCCAI	HBI	Mayo endoscopic score	Rutgeerts’ score	SES-CD	Histological score
Absent	<3	<5	0	0-1	0-2	0
Mild	3-5	5-7	1	2	3-6	1
Moderate	6-11	8-16	2	3	7-15	2
Severe	⩾12	>16	3	4	>15	3

Clinical response was defined as a decline of ⩾3 points in SCCAI or HBI as compared to baseline. Biochemical response was defined as a reduction of ⩾50% in FCP levels as compared to baseline. Endoscopic response was defined as a decline of 1 or more points in the endoscopic Mayo score, ⩾50% decline in SES-CD score or a decline of 1 or more points in the Rutgeerts’ score. Histologic remission was defined as a zero score in all collected biopsies.

### Protein isolation from human biopsies

During endoscopy at week 16, ileal and segmental colonic biopsies were collected (ascending colon, transverse colon, descending colon, sigmoid, and rectum). When possible, tissue was obtained from inflamed and non-inflamed mucosa, and stored in RNAlater^®^ at −20°C. For extraction of total protein content, biopsies were removed from RNAlater and lysed in 150 μl lysis buffer (10 mM Tris, 150 mM NaCl, 0.2% Triton X-100, and 2 mM EDTA) containing a cocktail of protease and phosphatase inhibitors, followed by 1 minute vortexing before and after 10 minutes incubation on ice.^35^ After centrifugation at maximum speed for 10 minutes at 4°C, the supernatant was collected and total protein content was measured using *DC* Protein Assay (Biorad^®^).

### Western blotting

For mouse samples, the whole colonic lysate was diluted 1:10 in Sample buffer containing 2-mercapto-ethanol and 15 µl of the diluted lysate was loaded on a 10% acrylamide gel. IDO1 expression was investigated using a mouse monoclonal antibody recognizing IDO1 (anti-IDO1 clone 8G11 antibody, *Merck-Millipore*). Monoclonal anti-β-tubulin antibody (clone AA2, *Sigma-Aldrich*) was used to confirm equal protein loading. Relative protein expression was quantified using an ImageQuant TL LAS4000 mini densitometer and the Analysis Toolbox software. IDO1 expression in human biopsies was evaluated on 25 µg of total protein lysate. Mouse monoclonal antibody anti-IDO1 (clone 10.1, *Merck-Millipore*) was used to detect IDO1 on human intestinal tissue. IDO1 expression was then reported as densitometric analysis score, performed with ImageQuant TL LAS4000 mini densitometer, per µg of total protein.^36^

### Kyn and Trp measurements

Mouse and human serum samples were kept frozen at −80°C until analysis. After protein precipitation, detection of Kyn and Trp concentrations was performed using a Perkin Elmer series 200 HPLC instrument (MA, USA).^17^ A Kinetex^®^ C18 column (250 mm × 4.6 mm, 5 μm, 100 Å; Phenomenex, USA), maintained at the temperature of 25°C and pressure of 1800 PSI, was used. A sample volume of 300 μl was injected and eluted by a mobile phase containing 10 mM NaH_2_PO_4_ pH 3.0 (99%) and methanol (1%) (Sigma-Aldrich, MO, USA), with a flow rate of 1 ml/min. Kyn was detected at 360 nm and Trp at 220 nm by an UV detector. The software TURBOCHROM4 was used for evaluating the concentration of Kyn and Trp in samples by means of a calibration curve. The detection limit of the analysis was 0.05 μM.

### Statistical analysis

Normally distributed data were presented as mean ± standard deviation (SD) and continuous data with a skewed distribution as median, and first and third quartile (Q1-Q3). Categorical data were presented as numbers and percentages. IBD-U patients were included in the UC group for analyses. After testing data distribution by D’Agostino & Pearson omnibus normality test, data that met normality were analyzed by two-tailed unpaired Student’s t-test. Mann-Whitney U test and Chi-square tests were used to evaluate differences in not-normally distributed data. DSS-colitis data were analyzed by two-way ANOVA with 2 variables, that is, time and treatment. One-way ANOVA analysis of variance—test for linear trend between mean and column number—was used to determine the significance of the trend between 3 or more groups. All analyses were performed in Graph Pad Prism version 6.0. A two-sided *P*-value of <.05 was considered statistically significant.

## Results

### The expression and activity of IDO1 decrease during chronicization of the colitis

In order to investigate the role of Trp metabolism in IBD, chronic colitis was modeled by subjecting mice to 2 separate cycles of 7-day DSS administration, followed by normal drinking water for 14 days (recovery phase) (Figure 1A). At the fifth day of DSS treatment, mice started to develop disease symptoms, including weight loss, diarrhea, and presence of blood in the stool. As opposed to healthy controls, animals receiving DSS exhibited a marked increase in DAI scores, which peaked at day +10 and decreased thereafter during the recovery phase in normal drinking water (Figure 1B). During the second cycle of DSS, a new increase in DAI score was observed, although this did not reach the same levels as observed in the first phase. At the end of the second DSS-cycle, colitis symptoms decreased (recovery phase) and, at day +42, the DAI neared the level of the control group (Figure 1B). On analyzing the body weight loss, we observed a marked reduction of body mass at day +10 post-DSS treatment (Figure 1C). At the end of the first recovery phase (day +15), mice significantly regained weight, indicating the relapsing-remitting trend of the DSS-induced chronic colitis. While IDO1 upregulation is well established in acute colitis,^37^ no clear evidence is available on the modulation of IDO1 levels during the chronic phase of DSS-colitis. In order to analyze IDO1 levels throughout the course of the DSS chronic colitis model, mice were euthanized at day 7, 14, 21, 28, 35, or 42 after first DSS administration, and IDO1 expression was analyzed. As previously demonstrated,^25,37,38^ the expression of IDO1 in the intestinal mucosa was strongly increased in the acute phase of DSS-colitis (day +7); however, the protein level returned rapidly to basal levels during the recovery period (Figure 1D and Supplemental Figure S1). Corresponding with reduced disease activity scores during re-challenge of mice with a second cycle of DSS, IDO1 levels stabilized during the second DSS challenge, and a further reduction of IDO1 expression was found during the second recovery period (Figure 1D). The systemic catalytic activity of IDO1 was evaluated by measuring serum levels of Kyn and Trp. In accordance with the variation of IDO1 protein expression in the colon, we found that the serum Kyn/Trp ratios increased at day +7 of DSS challenge (ie, in the acute stage of the disease), and decreased during recovery. In line with the inflammation score, a second DSS challenge was associated with a weaker increase in IDO1 activity compared to day +7, which again was restored during the second recovery phase (Figure 1E).

Overall, these data demonstrate that the local expression (ie, in the colon of mice exposed to DSS) as well as the systemic activity of IDO1 increases during the first stage of inflammation, likely with the aim to counterbalance the strong immune activation. However, in the following stages of chronic colitis IDO1 activity decreases along with the disease severity.

### Enhanced IDO1 activity during the chronic phase protects intestinal mucosa from DSS-insult resulting in modest amelioration of disease symptoms

**Figure 2.**
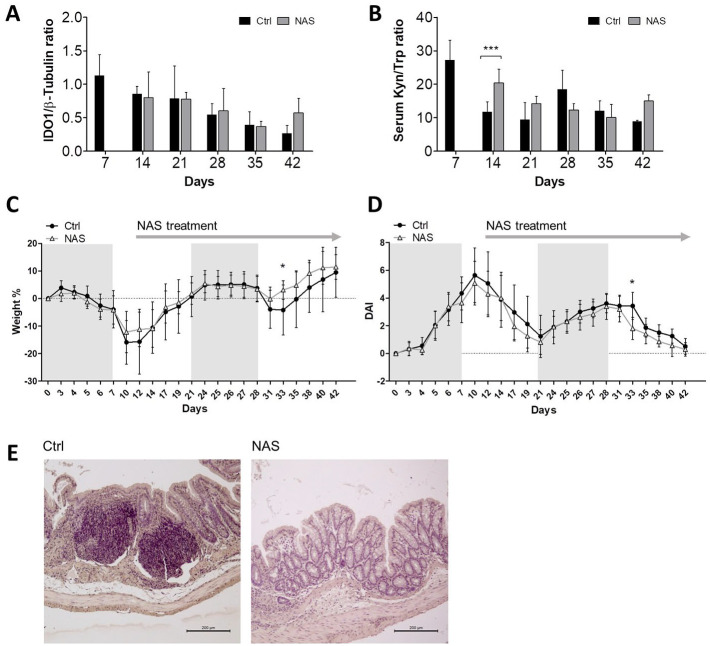
Modulation of IDO1 activity and expression with NAS has weak therapeutic effects. (A) Lysates from colons of mice subjected to alternative cycles of DSS and normal drinking water and treated with either vehicle (Ctrl) or N-acetyl serotonin (NAS) (10 mg/kg, 3 times per week, starting from day +12) were analyzed by Western blot analysis using an antibody against IDO1, with anti-β-tubulin antibodies used to control for protein content. Samples were analyzed at the indicated time after DSS challenge, mean of n = 3 colon samples is shown. (B) Ratio of kynurenine (Kyn) and Tryptophan (Trp) levels as measured by HPLC (mean ± SD) in serum samples of mice treated as in (A). Three samples were analyzed at each indicated time after DSS challenge. (C) Body weight change in mice subjected to DSS-induced chronic colitis and treated with vehicle (Ctrl) or NAS. Values are reported as percentage of body weight change calculated using the formula [(Weight at day X/Weight at day 0) × 100]. (D) Disease activity index (DAI) scores were measured daily by combining scores of weight loss, stool consistency, and presence of fecal blood. Values in C and D are reported as mean ± SD from 12 mice/group of 2 independent experiments. (E) Representative images of histological sections of colons taken at day 42 from mice exposed to DSS and then treated with vehicle alone (Ctrl) or NAS. Panels are representative of the 24 mice investigated in 2 independent experiments. Data in (A-D) were analyzed with two-way ANOVA, followed by post hoc Bonferroni test. Ctrl, mice receiving vehicle alone. NAS, mice treated with NAS. *P < .05 and ***P < .001.

It has been shown that the pharmacologic modulation of IDO1 activity can affect the symptoms of acute colitis.^25-27^ Therefore, we investigated whether the modulation of IDO1 activity could affect the late phase of colitis. To this purpose, mice were treated starting from day +12 with NAS, previously demonstrated to enhance IDO1 activity in DCs through positive allosteric modulation of IDO1.^21,39^ Although the NAS treatment did not modify the expression of IDO1 protein in the colon (Figure 2A), it increased the systemic Kyn/Trp ratio at day +14 (Figure 2B), confirming the positive modulation of IDO1 catalytic activity rather than its protein expression. Interestingly, the enhancement of IDO1 activity improved the recovery rate 5 days after DSS withdrawal (day +33), resulting in a significantly increased weight (Figure 2C) and a decreased DAI score (Figure 2D) of NAS-treated mice as compared to the control. Although NAS showed a modest effects on the clinical course of the disease, evaluation of pathological reports at day +42 of the intestinal mucosa of NAS-treated mice revealed a defined conformation of the intestinal villi and modest immune infiltrate, compared to the control mice which experienced a total destruction of the intestinal mucosal architecture with a diffuse inflammatory infiltrate (Figure 2E). Overall, these data suggest that increased IDO1 enzymatic activity during a flare, while not affecting the primary disease course, may ameliorate the inflammation of the intestinal mucosa, preventing the destruction of the tissue architecture.

### IDO1 expression and activity correlates with the severity of mucosal inflammation

Next, we investigated whether IDO1 is also modulated in human disease during chronic inflammation. IDO1 expression and catalytic activity were assessed in human biopsy samples collected from IBD patients. We prospectively included patients starting treatment with either the anti-α4β7 integrin antibody VDZ (inhibiting lymphocyte trafficking to the gut mucosa), or the anti-p40 antibody UST (blocking the IL12/IL23 signaling involved in IBD pathogenesis).^40^ Overall, samples from 93 patients affected by IBD with a median disease duration of 14.3 years (IQR 7-18) at week 16 of the pharmacological treatment (namely, n = 37 treated with VDZ and n = 56 treated with UST) were analyzed (Table 2). In total, 87/93 (94%) patients were previously exposed to ⩾1 anti-TNFα drugs before starting VDZ or UST, of whom 79/87 (91%) were anti-TNFα-refractory, defined as having primary non-response or secondary loss of response, and 8/87 (9%) stopped anti-TNFα because of adverse effects. Furthermore, 28/56 UST treated patients (50%) were previously exposed to VDZ therapy, reflecting the refractory, chronic nature of this cohort of patients. In 53 patients (57%), VDZ or UST induction was combined with corticosteroid induction therapy, which was continued until week 16 in 29 (55%) of these patients. Moreover, 15 patients (16%) were on concomitant immunomodulatory therapy and 6 patients (6%) on 5-aminosalicylates during induction.

**Table 2.** Patient baseline characteristics.

	N = 93
Female, n (%)	56 (60)
Median age, years (25-75th)	40 (27-52)
Smoking, n (%)	9 (24)
Median disease duration, years (25-75th)	14 (7-18)
Diagnosis, n (%)	
UC	15 (16)
CD	78 (84)
CD disease location, n (%)	
L1 ileal	8 (10)
L2 colonic	8 (10)
L3 ileocolonic	62 (80)
+L4 upper GI disease	9 (11)
CD disease behavior, n (%)	
B1	29 (37)
B2	40 (51)
B3	9 (11)
Perianal disease	14 (18)
UC disease location, n (%)	
E2	4 (27)
E3	11 (73)
Previous intestinal resection, n (%)	49 (52)
Anti-TNFα exposed, n (%)	
naive	6 (6)
1	20 (22)
⩾2	67 (72)
Anti-TNFα refractory disease, n (%)	79 (94)
Anti-TNFα cessation due to side-effects, n (%)	8 (6)
Concomitant steroid induction therapy, n (%)	53 (57)
Concomitant IBD medication, n (%)	
Immunomodulator	15 (16)
5-ASA	6 (6)

At week 16, 37 patients (40%) showed a clinical response and 36/86 patients (42%, data unavailable for 7 patients) showed biochemical response. The week 16 endoscopic response rate was 45% (42/93 patients). Histological assessment was available in 70/93 (75%) patients, of whom 22 (31%) showed histological remission at week 16.

**Figure 3.**
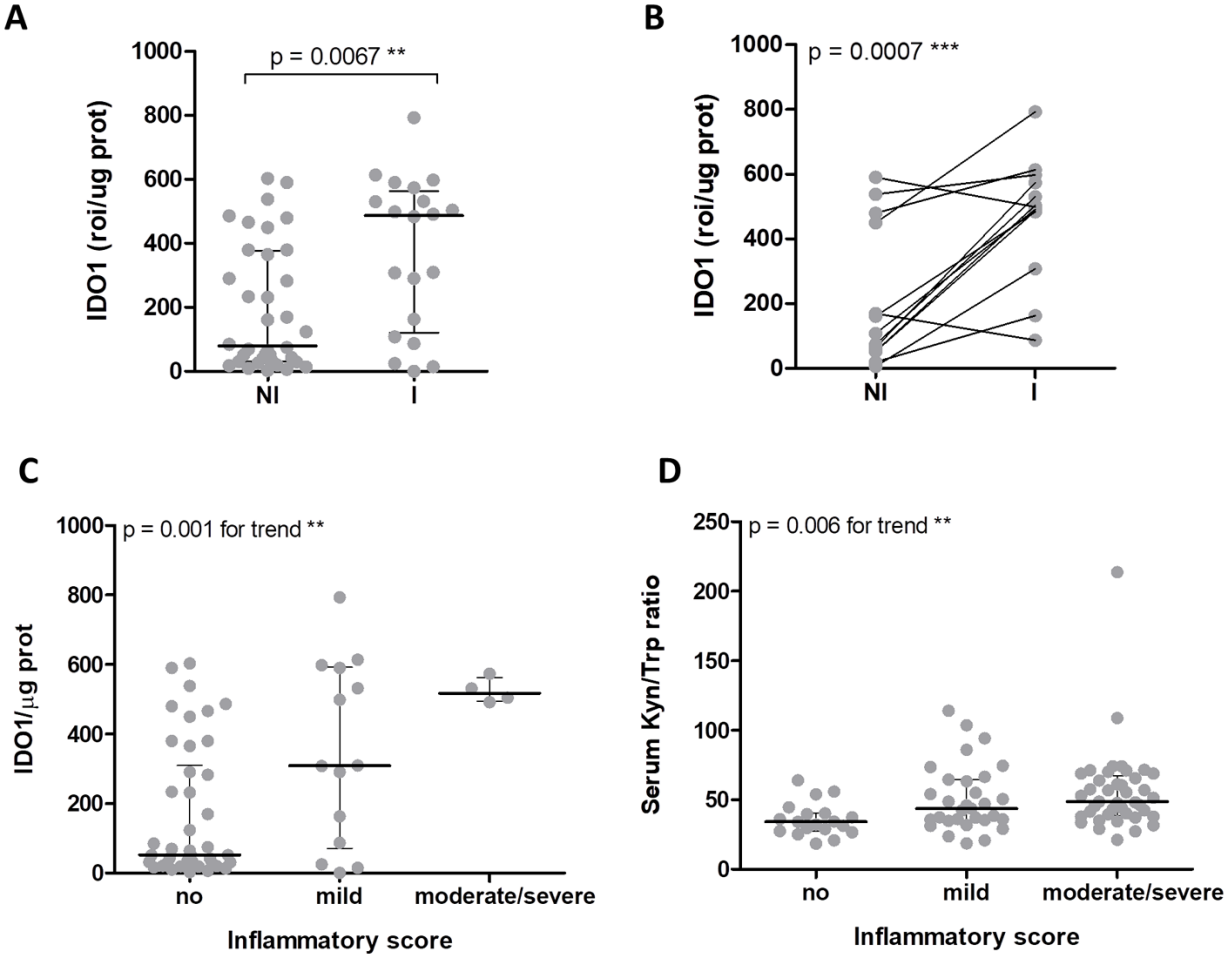
Mucosal inflammation at week 16 is associated with increased IDO1 expression and catalytic activity. (A-C) IDO1 protein expression was evaluated on a total of 56 protein lysates obtained from 24 biopsies from VDZ-treated patients, and 32 biopsies from UST-treated patients. IDO1/µg protein ratio was calculated by densitometric quantification of the specific IDO1 band detected by Western blot analysis and normalized for total protein content. (A) IDO1 protein expression in tissue lysates obtained from endoscopically inflamed regions was compared to IDO1 levels in biopsies taken from non-inflamed regions. (B) Biopsies from inflamed and non-inflamed mucosal regions from the same patient were compared. (C) Biopsies obtained from IBD patients were stratified in no, mild, or moderate/severe inflammation according to histological assessment. (D) Comparison of serum Kynurenine (Kyn) to Tryptophan (Trp) ratios as measured by HPLC between patients stratified according to their endoscopic score in no, mild, or moderate/severe inflammation. Median values and the Q1-Q3 range are indicated by lines and whiskers. One-way ANOVA analysis of variance—test for linear trend between mean and column number—was used to determine the significance of the trend in (C) and (D).

IDO1 protein expression was evaluated on a total of 56 protein lysates obtained from 24 biopsies from VDZ-treated patients, and 32 biopsies from UST-treated patients. Biopsies taken from endoscopically inflamed regions showed a significantly higher IDO1 protein expression as compared to biopsies taken from non-inflamed regions (Figure 3A examples shown in Supplemental Figure S2). This was also apparent when matched biopsies from inflamed and non-inflamed mucosal regions from the same patient were compared (Figure 3B), suggesting that IDO1 expression rises locally during disease activity. By stratifying the biopsies based on their histological score, a strong correlation between IDO1 expression and severity of local inflammation was found (*P* = .003 for trend, Figure 3C examples shown in Supplemental Figure S2). The systemic catalytic activity of IDO1 was also evaluated by measuring Kyn/Trp ratio in the sera of both VDZ- and UST-treated patients (n = 93). Kyn/Trp ratios were comparable between CD and UC patients (*P* = 1.0, data not shown) and the type of biological treatment did not affect the systemic IDO1 activity (*P* = .11, data not shown). Stratification of patients based on their most severe histological score, showed that the Kyn/Trp ratio was lowest in sera obtained from patients without inflammation and increased in patients with mild to moderate/severe endoscopic inflammation (*P* = .02; Figure 3D). Thus, IDO1 protein expression locally increases with the severity of disease, translating into a systemic alteration of the Kyn/Trp ratio.

### Vedolizumab/Ustekinumab treatment response is associated with reduced IDO1 expression and activity levels

**Figure 4.**
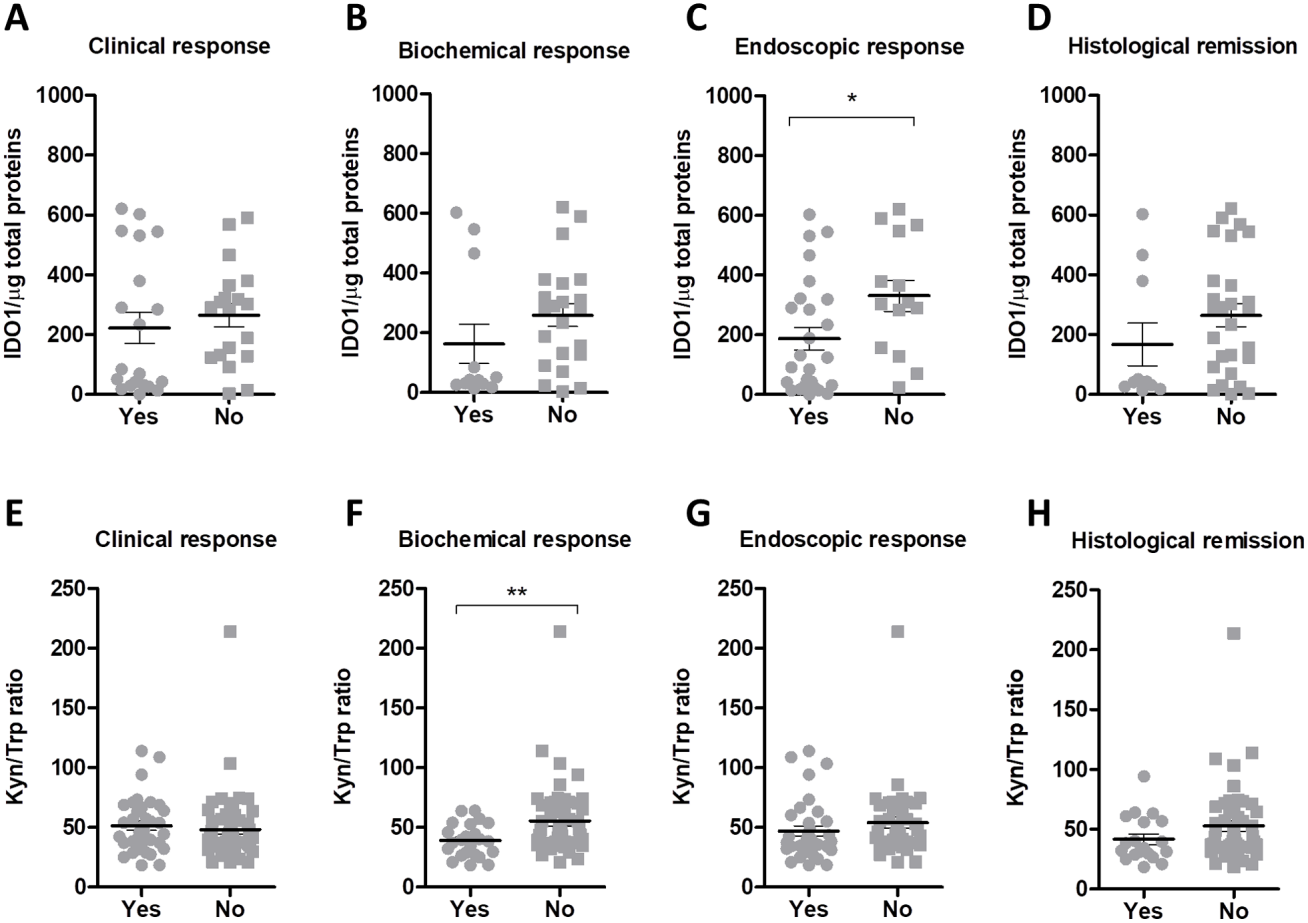
Reduced IDO1 expression and catalytic activity is associated with treatment response. (A-D) Comparison of tissue IDO1 levels between patients who did (Yes) or did not (No) show clinical response (A), biochemical response (B), endoscopic response (C), and histological remission (D). IDO1 expression is reported as densitometric analysis per µg of total protein. Median values and the Q1-Q3 range are indicated by lines and whiskers. (E-H) Comparison of serum Kyn/trp ratio between patients showing (Yes) or not showing (No) clinical response (E), biochemical response (F), endoscopic response (G), or histological remission (H). Median values and the Q1-Q3 range are indicated by lines and whiskers. After testing data distribution by D’Agostino & Pearson omnibus normality test, data were analyzed by two-tailed unpaired Student’s *t*-test. *P < .05 and **P < .01.

On analyzing the correlation between IDO1 protein expression and clinical response to the pharmacological treatment, no differences were observed in the median IDO1 tissue levels between patients with or without clinical response (77.0 vs 290.3, *P* = .27; Figure 4A). IDO1 levels in biopsies from patients showing biochemical response were lower, albeit not significantly, compared to non-responders (42.1 vs 283.5, *P* = .09; Figure 4B), and patients achieving endoscopic response to the therapy showed significantly lower IDO1 levels as compared to those that did not respond (107.5 vs 306.0, *P* = .02; Figure 4C). Association between median IDO1 tissue levels and histological remission did not achieve statistical significance (42.1 vs 282.7, *P* = .2; Figure 4D).

On investigating the association between serum Kyn/Trp ratio and treatment response, no significant correlation was found for clinical response (46.9 vs 41.9, *P* = .3; Figure 4E). However, patients achieving biochemical response showed a lower Kyn/Trp ratio compared to non-responders (38.43 vs 47.21, *P* = .007; Figure 4F). The same trend, although not significant, was found for endoscopic response (37.87 vs 47.21, *P* = .08; Figure 4G) and histological remission (34.22 vs 42.36, *P* = .09; Figure 4H).

Taken together, these data demonstrate that a reduction in IDO1 levels follows disease regression in IBD patients.

## Discussion

In the last decades, several studies have been conducted on different mouse models in order to elucidate the role of IDO1 in the pathogenesis of IBD, but contradictory results have been described. In light of its immunosuppressive role in many physiological processes (eg, pregnancy, transplantation settings, allergy, autoimmunity), many studies have suggested that the increase of IDO1 expression occurring in response to pro-inflammatory signals could be viewed as part of a negative feedback loop that helps to limit the inflammatory responses.^22^ However, others suggested that IDO1 may have a pro-inflammatory role in the context of IBD since *Ido1*^−/−^ mice show milder symptoms in colitis models.^30,41^ A possible explanation for why *Ido1*-deficient mice are more protected from colitis has been recently reported by Shin et al^41^ They showed that *Ido1* deficiency affects intestinal microbiota profiles, increasing intestinal bacteria that use tryptophan preferentially to produce indolic compounds which are stronger agonists of AhR compared to Kyn and mediate immunoregulatory effects in the gut.^42^ Another possible mechanism was previously provided by Metghalchi et al^43^ who observed a marked increase of IL-10 levels in *Ido1*^−/−^ mice, associated with a protective effect both against colitis and atherosclerosis. However, inflammation in humans is the result of an imbalance in the complex interaction between pro- and anti-inflammatory pathways as well as microbial alterations, and while loss of *IL10* is seen only in some very early onset monogenetic IBD cases, loss of IDO1 has not been reported. Furthermore, the complexity of IDO1 and its metabolites in inflammatory processes should be underlined. For instance, it has been recently demonstrated that some tryptophan-derived metabolites exert a protective role following AhR activation, while others appear to have deleterious effects.^42,44^ Furthermore, activation of AhR by Kyn can shift the balance of other important AhR-mediated immunoregulatory pathways and has been associated with gut dysbiosis.^42,45^ It should also be considered that the discrepancies regarding the role of IDO1 in IBD may be influenced by the mouse model chosen for the study since different immunological mechanisms are involved depending on how colitis is induced.^46,47^ For instance, while trinitrobenzene sulfonic acid (TNBS) induces colitis in a specific Th1 cell-dependent manner, the DSS-induced colitis model depends on innate immune activation upon colonic epithelial barrier destruction to mimic colitis, which may have different IDO1 dependency.^48,49^ Nevertheless, our understanding of the role of IDO1 in IBD is hampered by the fact that most of the published studies have been conducted in models of acute colitis, which only partially reproduce the complex mechanism of chronic diseases such as UC and CD.^25-27,30-32,41^ To the best of our knowledge, our study is the first to investigate IDO1 in a chronic colitis model. Our results demonstrate that IDO1 expression and activity closely follow disease activity, with a rapid increase during the acute phase of the colitis, and lower levels during recovery and a second DSS challenge. While this could theoretically suggest that IDO1 is a mediator of disease activity, it is more likely that upregulated IDO1 levels during an inflammatory challenge reflect a “failed” immunoregulatory response. Our data suggest that IDO1 activity contributes to the resolution of the disease, as treatment of mice with the IDO1 agonist NAS speeds up the recovery phase after the second DSS cycle. Interestingly, the positive effects of the enhanced IDO1 activity on disease course became apparent at the day +33, when the Kyn/Trp ratio was no longer affected by NAS treatment. This could either mean that IDO1-mediated effects take a long time to manifest, or that local mucosal IDO1 activity changes were no longer reflected by systemic measurements. Indeed, our data in human IBD patients indicate that local intestinal IDO1 levels may vary, depending on the severity of inflammation (Figure 3A). Although from our experiments the increase in the catalytic activity of IDO1 following treatment with NAS is evident, we cannot exclude that IDO1-independ effects may contribute to its protective effect against colitis.^11,42^ NAS is an immediate precursor of melatonin which has been shown to have the ability to suppress inflammatory responses.^50^ NAS has also been shown to have the ability to activate the brain-derived neurotrophic factor (BDNF) receptor,^51^ implicated in inflammatory bowel pathophysiology.^52,53^

Several studies in patients affected by CD and UC showed increased expression of IDO1 mRNA and protein in both the lamina propria and epithelium during active IBD compared to controls.^23,24,54^ These findings are consistent with those of Nikolaus et al^3^ who showed a significant increase in Kyn/Trp ratio in patients with active disease compare to inactive disease or healthy controls.

The current study confirmed this association, showing that IDO1 protein level and the serum Kyn/Trp ratio significantly correlate with endoscopic and histological disease activity. In addition, IDO1 activity was assessed in relation to the pharmacologic response to both VDZ and UST immunotherapeutic drugs. Discrepant observations have been previously reported in the literature about the expression of IDO1 in the mucosa and the response to the biological infliximab (namely, an anti-TNFα antibody). While Wolf et al^23^ showed a reduced IDO1 expression in the mucosa, Zhou et al^24^ showed no IDO1 modulation during infliximab treatment. Both these studies analyzed IDO1 protein expression by immunochemistry in few tissue samples, only 6 and 5 severe CD cases, respectively. Our study included a larger number of IBD patients (n = 40) and extended the analysis of IDO1 expression and activity to further immunotherapy (namely, the anti-α4β7 integrinVDZ and the anti-IL12/23 UST). Overall, our observations indicate that the response to the biological treatment is associated with a reduced IDO1 protein level in the mucosa of IBD patients, suggesting that IDO1 expression is affected by the local inflammation patterns and could be important for the resolution of IBD disease.

We acknowledge several limitations to our study. In the murine model of the disease, we were unable to compare inflamed versus non-inflamed tissues within the same colon, which, as our human data suggest, might be relevant. In the human disease, we were unable to collect patient’s biopsies at the baseline, precluding the analysis of IDO1 expression and activity at the baseline. Interestingly, a lower expression of IDO1 is observed in immune cells from IBD patients compared to healthy controls^54^ and reduced Kyn levels have been measured in colonic explants from non-lesional biopsies from IBD patients,^23^ with lower Kyn/Trp ratios in non-lesional UC patients, suggesting that a loss of tolerance caused by reduced IDO1 levels could underlie the disease. In conclusion, the current study suggests that, despite the complexity of intestinal pathophysiology, Trp metabolism still plays an important role in resolution of disease, and that chronic mouse models may provide novel insight into disease pathology. Moreover, mucosal levels of IDO1 as well as systemic catalytic activity are significantly decreased in patients achieving endoscopic response at week 16, suggesting that IDO1 enzymatic activity may hold promise as biomarker for disease and treatment monitoring in IBD. However, further research is needed to better clarify the issue.

## Supplemental Material

sj-docx-1-try-10.1177_11786469231153109 – Supplemental material for Modulation of Indoleamine 2,3-Dioxygenase 1 During Inflammatory Bowel Disease Activity in Humans and Mice Click here for additional data file. Supplemental material, sj-docx-1-try-10.1177_11786469231153109 for Modulation of Indoleamine 2,3-Dioxygenase 1 During Inflammatory Bowel Disease Activity in Humans and Mice by Elisa Proietti, Renske W.M. Pauwels, Annemarie C. de Vries, Elena Orecchini, Claudia Volpi, Ciriana Orabona, Maikel P. Peppelenbosch, Gwenny M. Fuhler and Giada Mondanelli in International Journal of Tryptophan Research
