# Supplementary material for: Modulation of Indoleamine 2,3-Dioxygenase 1 During Inflammatory Bowel Disease Activity in Humans and Mice
Source: Int J Tryptophan Res. 2023 Feb 9;16:11786469231153109. doi: 10.1177/11786469231153109 (PMC9926376; doi:10.1177/11786469231153109)
Supplement: sj-docx-1-try-10.1177_11786469231153109 – Supplemental material for Modulation of Indoleamine 2,3-Dioxygenase 1 During Inflammatory Bowel Disease Activity in Humans and Mice [file sj-docx-1-try-10.1177_11786469231153109.docx]

***Supplementary material***

*Supplementary figure 1: IDO1 protein expression on murine colon lysates.*


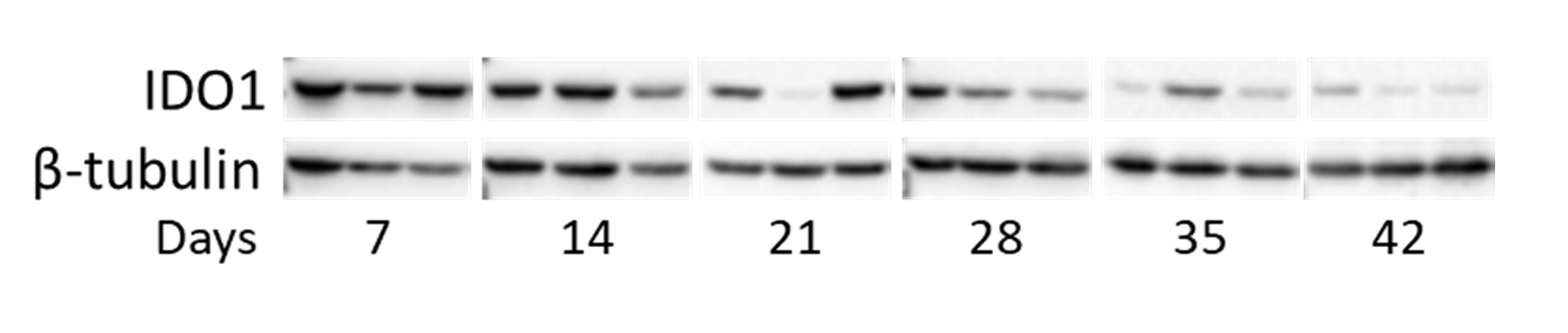


Representative images of immunoblot analysis of colonic mucosal tissue lysate collected at the indicated time-points from mice subjected to alternative cycles of DSS and normal drinking water. IDO1 was detected using a mouse monoclonal antibody recognizing IDO1 (anti-IDO1 clone 8G11 antibody, *Merck-Millipore*). Monoclonal anti-β-tubulin antibody (clone AA2, *Sigma-Aldrich*) was used to confirm equal protein loading.

*Supplementary figure 2: IDO1 protein expression on human colon lysates.*


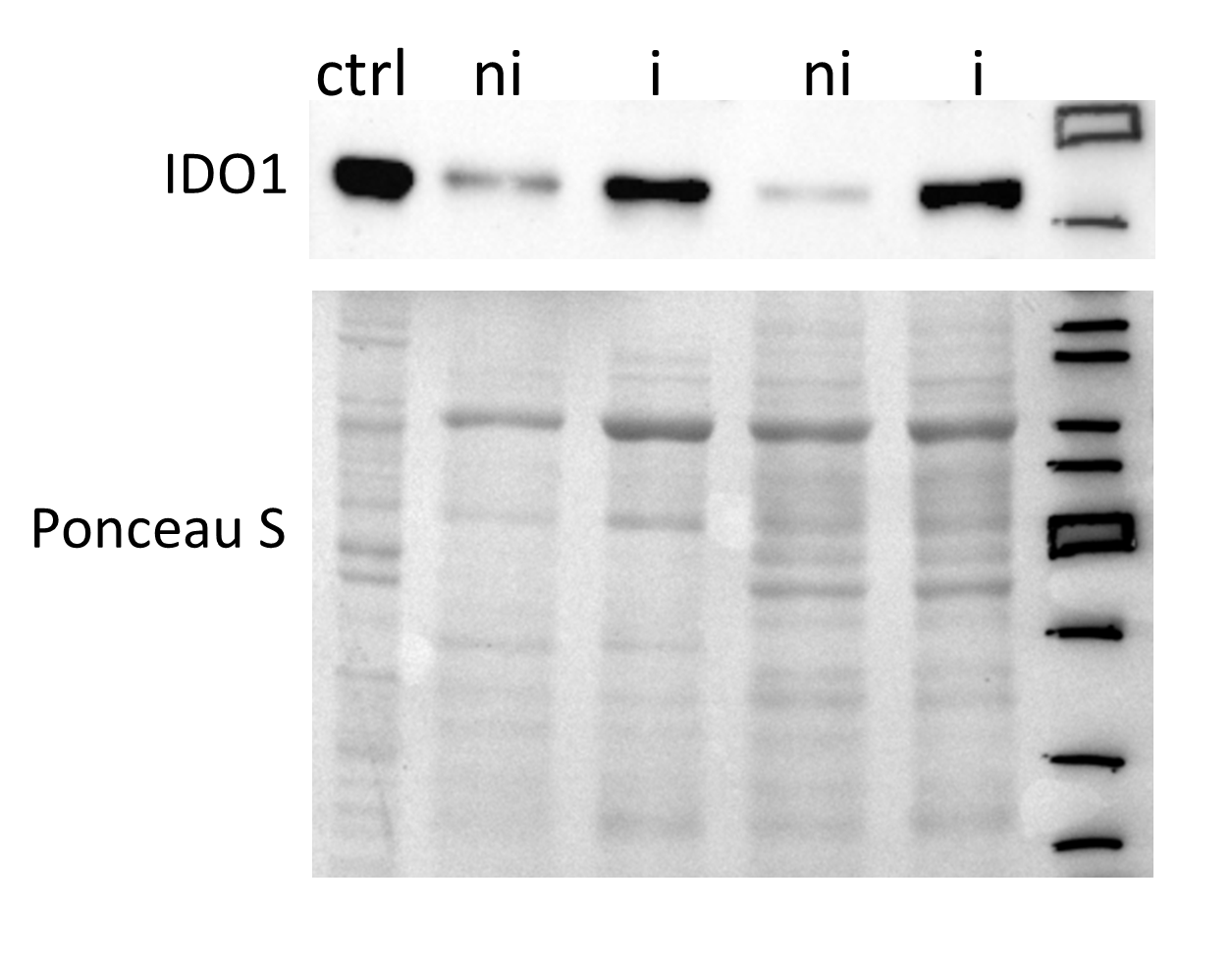


Representative image of immunoblot analysis on colonic mucosal tissue lysate from IBD patients. IDO1 was detected using anti-IDO1 monoclonal antibody clone 10.1 (Millipore). Ponceau S staining of the corresponding nitrocellulose membrane is shown. ctrl, positive control sample. i, inflamed tissue. ni, non inflamed tissue.
